# ABA perception is modulated by membrane receptor-like kinases

**DOI:** 10.1093/jxb/erz531

**Published:** 2020-02-19

**Authors:** Dorota Konopka-Postupolska, Grazyna Dobrowolska

**Affiliations:** Institute of Biochemistry and Biophysics, Polish Academy of Science, Warsaw, Poland

**Keywords:** ABA-induced stomatal closure, ABI1, abscisic acid, BAK1, OST1, RPK1, sequential complex formation

## Abstract

This article comments on:

**Shang Y, Yang D, Ha Y, Shin H-Y, Nam KH**. 2020. RPK1 and BAK1 sequentially form complexes with OST1 to regulate ABA-induced stomatal closure. Journal of Experimental Botany 71, 1491–1502.

**In this issue of the *Journal of Experimental Botany*, Shang *et al.* (2020) characterize the possible role of membrane receptor-like protein kinase 1 (RPK1) in abscisic acid (ABA) signaling. The authors show that upon ABA accumulation, RPK1 can form a complex and possibly regulate the ABA-mediated activation of the OST1 kinase—the major regulator of ABA-induced responsive pathways. *Arabidopsis thaliana* mutants that lack functional RPK1 are ABA resistant and show increased water loss, whereas overexpressing plants have an increased tolerance to drought stress (Osakabe *et al.*, 2010). The existence of a positive feedback regulation loop among RPK1, calmodulin 4, and NADPH oxidase F (respiratory burst oxidase homolog F, RBOHF) at the transcriptional level was reported (Koo *et al.*, 2017). Thus, RPK1 may constitute a link to the plasma membrane perception of ABA.**

In previous research, the same group showed that ABA signaling is modulated by another receptor-like kinase, Brassinosteroid Insensitive 1 (BRI1) Associated Kinase 1, BAK1. It has been shown that BAK1 interacts with and phosphorylates OST1, possibly affecting in this way its activity. BAK1 is regarded as a master regulator/co-receptor of the activity of several membrane receptor kinases/receptor-like kinases (RKs/RLKs), such as BRI1, flagellin receptor (FLS2), and EF-Tu RECEPTOR (EFR) regulating plant growth, development, and stress responses. Shang *et al.* (2020) showed that OST1 can form a complex with RPK1 with different kinetics compared with BAK1. It suggests that the basic signaling pathway mediating stomatal closure can be modulated by interactions of OST1 with different membrane leucine-rich repeat receptor kinases. It can allow the fine-tuning of ABA signaling in guard cells by multiple networks.

ABA is a plant hormone most widely known for its capability to activate the response to environmental stresses, especially those connected with dehydration. Besides plants, ABA is synthesized in fungi (Nambara and Marion-Poll, 2005) and has been found to mediate physiological effects in a wide range of phylogenetically unrelated organisms, from unicellular prokaryotes (cyanobacteria), through simple invertebrates (such as sea sponges) up to humans (Wasilewska *et al.*, 2008). Moreover, there are some similarities in ABA response mechanisms (e.g. intracellular receptors) between evolutionarily distant organisms, which suggests that ABA signaling is evolutionarily ancient (Wasilewska *et al.*, 2008; Shinozawa *et al.*, 2019).

## Physiological effects of ABA—puzzling diversity of biosynthesis and the basic pathway of intracellular perception

The vast majority of studies conducted so far focused on the physiological effects of ABA on plants. Endogenous ABA regulates almost all aspects of plant life, beginning from sprouting up to senescence. During growth, ABA determines organ and body size and root growth, modulates plant metabolism, and controls fertility (Harris, 2015). During stresses, such as drought, salt, and, to a lesser extent, low temperature, it protects plants acting as a key regulator of adaptation to biotic and abiotic stresses. Functional diversity, which is inherent to ABA signaling, requires a very sophisticated and spatiotemporally specific detection and reaction system. In the last few years, significant progress has been made towards understanding the mechanisms contributing to ABA biosynthesis, transport, perception, and identification of intracellular targets. However, apart from its existence, relatively little is known about ABA sensing at the plasma membrane, and understanding of the interplay between independent sensing units remains a matter for future research.

One of the most thoroughly studied ABA signaling pathways is found in guard cells, leading to stomatal closure during soil dehydration or lowering of air humidity. For many years it has been known that phosphorylation and dephosphorylation of proteins are essential for this process (Yang *et al.*, 2017). In protoplasts of *Arabidopsis thaliana* (Arabidopsis) guard cells more than half of the kinase genes identified in the Arabidopsis genome (689 out of 1019) and all genes encoding phosphatase catalytic subunits (Wang *et al.*, 2007) are expressed. Plants with defects in certain phosphatases or kinases display ABA-hypersensitive or ABA-insensitive phenotypes. Mutations in a specific group of phosphatases, specifically the A clade of PP2C (ABI1, ABI2, and HAB1), result in ABA hypersensitivy manifested, among others, by enhanced tolerance to drought. In turn, a kinase from the sucrose non-fermenting-1 (SNF1)-related protein kinases 2 family (SNRK2)—SNRK2.6, also called open stomata 1 (OST1)—was identified as a critical positive regulator of ABA signal transduction in guard cells and its inactivation resulted in complete inhibition of ABA-induced stomatal closure (Acharya *et al.*, 2013). In the absence of ABA, OST1 activity is inhibited by dephosphorylation and direct physical interaction between kinase and PP2C phosphatases (Ng *et al.*, 2014). When ABA is present, this complex dissociates and OST1 is released from phosphatase inhibition (Box 1). This results in activation of the kinase via autophosphorylation of Ser175 in the kinase activation loop. Dissociation of the kinase–phosphatase complex in the presence of ABA results from binding of phosphatase to the holo form of the soluble ABA receptor (PYR/PYL/RCAR). Once it has bound to a ligand, the receptor undergoes a conformational change that results in the creation of a platform for tight binding and inactivation of PP2Cs (Ng *et al.*, 2014). Therefore, the shortest basic ABA-sensing pathway identified in guard cells comprises PYR/PYL/RCAR receptor (ABA receptor), ABI1/HAB1 (a phosphatase), OST1 (a protein kinase), and a target protein. Among downstream targets of free OST1 in guard cells, there is the slow anion channel-associated 1 (SLAC1), quickly activating anion channel (QUAC1), a major anion channel in guard cells, potassium inward rectifying channel (KAT1s), and RBOHF (Arnaud and Hwang, 2015). However, experimental results suggest that ABA signaling in guard cells is also regulated by several additional pathways initiated at the cellular membrane by other hormones or environmental factors (Cutler *et al.*, 2010).

Box 1. **Plant membrane perception**

Plant genomes contain the greatly expanded monophyletic gene family that code for receptor-like protein kinases (RPKs/RLKs), structurally and functionally related to animal receptor kinases from the Pelle family. In individual species, RLKs account for ~2% of the total number of coding genes (i.e. ~610 in *Arabidopsis thaliana* and 1100 in *Oryza sativa*). They establish a surveillance system for the detection of environmental factors as well as perceiving diverse internal signals to orchestrate growth and development, and to control self-incompatibility. The typical RLKs consist of an N-terminal extracellular ‘receptor’ domain, a single transmembrane domain, and a C-terminal intracellular domain with protein kinase activity. According to the structure of their extracellular domains, RLKs are classified into several subfamilies. The best recognized is a subfamily containing extracellular leucine-rich repeat motifs (LRR-RLK) whose members are BRASSINOSTEROID INSENSITIVE 1 (BRI1), BRI1-associated kinase 1 (BAK1), CLAVATA1, PEP RECEPTOR 1 (PEPR1) and PEPR2 pattern recognition receptors (PRRs), FLAGELLIN SENSING2 (FLS2), and EF-Tu receptor (EFR). Broadly, they are involved in the perception and initiation of signaling. Another group are the cysteine-rich receptor-like kinases (CRKs) (44 in Arabidopsis) with the extracellular cysteine-rich motif of unknown function (C-X8-C-X2-C, DUF26) available for extracellular signal perception. The precise role of the DUF26 domain is unknown, but it was suggested that it is involved in redox regulation and protein–protein interaction (Bourdais *et al.*, 2015). Some CRKs were proposed to assist in ROS perception during the response to biotic and abiotic environmental factors (Bourdais *et al.*, 2015). Approximately 25% of Arabidopsis RLKs do not have an ectodomain (and, in some cases also lack a transmembrane domain) and thus are called the receptor-like cytoplasmic protein kinases (RLCKs) (Liang and Zhou, 2018). RLCKs are often functionally and/or physically associated with RLKs and can transmit their activation downstream via transphosphorylation (Liang and Zhou, 2018).

The only ABA receptor proteins identified so far are localized intracellularly but the hormone is also perceived at the plasma membrane, although no bona fide membrane ABA receptor has been identified. *Arabidopsis thaliana* plants with a functional knockout of RPK1 are more resistant than the wild type to exogenous ABA. It was shown that RPK1 together with calmodulin 4 and NADPH oxidase F (respiratory burst oxidase homolog F, RBOHF) mediate the transient accumulation of H_2_O_2_ and trigger age-dependent cell death (Koo *et al.*, 2017). Nevertheless, the molecular mechanisms of RPK1 activation and its downstream targets remain elusive.

Activation of certain RLKs by ligand binding results in their association with their respective co-receptors and the formation of heteromeric complexes at the plasma membrane. During plant immunity, activated FLS2 and EFR recruit BAK1 and form active receptor complexes. Ultimately this leads to the activation of the appropriate RLCKs, such as BOTRYTIS-INDUCED KINASE 1 (BIK1) or PBS1-LIKE1 (PBL1). Organization of RLKs into heterooligomeric protein complexes can be a universal feature of such signaling, and BAK1 was shown to function as a co-receptor in different responses, such as innate immunity and brassinosteroid signaling. When RPK1 from *A. thaliana* was overexpressed in rice cells, it formed clusters in the cell membrane (Shi *et al.*, 2014).

Once activated, RLCKs dissociate from the receptor complexes and, in turn, interact with and phosphorylate their targets, such as RBOHs. Among the RBOH isoforms, RBOHD is responsible to the greatest extent for the production of apoplastic H_2_O_2_ during bacteria-induced stomatal closing, whereas RBOHF is the main isoform that is involved in ABA-induced reactive oxygen species (ROS) production and ABA-mediated stomatal closure (Arnaud and Hwang, 2015). Accumulation of ROS in the apoplast is both necessary and sufficient to induce stomatal closure. It induces ROS-dependent Ca^2+^ influx which, in turn, activates a positive feedback loop mediated by different calcium-dependent kinases that activate by phosphorylating RBOHD and RBOHF to amplify ROS production (Arnaud and Hwang, 2015).

So far, it has been accepted that ABA activates stomatal closure by the action of OST1 on RBOHF and SLOW ANION CHANNEL-ASSOCIATED 1 (SLAC1), the major executor of stomatal closure (Arnaud and Hwang, 2015). However, the physical interactions between OST1 and RPK1 shed new light on this process. OST1-induced RBOHF activity results in the accumulation of ROS in the apoplast, which can result in the activation of CRKs. In particular, CRKs (CRK6, CRK7, CRK8, CRK10, and CRK15) were shown to be involved in ROS sensing and proper response to extracellular ROS (Bourdais *et al.*, 2015). Moreover, some CRKs were found to be associated with FLS2 or BAK1 (Kimura *et al.*, 2017) and, together with BIK1 and RBOHD/RBOHF, may form a positive activation loop that enhances ROS burst and leads to the promotion of stomatal immunity (Lee *et al.*, 2017). The same set of CRKs is also involved in ABA-mediated responses. Therefore, it is tempting to speculate that RPK1 could form a complex with BAK1 and CRKs during ABA-mediated responses. Activation of RPK1 could happen by transphosphorylation in a complex with CRK or with mobile signaling molecules that have not yet been identified. For example, RPK1 mediates signaling by a small peptide, CLE, in the pathway that controls root growth (Racolta *et al.*, 2018). The activated receptor complex containing RPK1 can associate with OST1 and modify its activity by phosphorylation.

## Perception of ABA at the plasma membrane

Plants perceive extracellular signals at the plasma membrane by receptor-like kinases (RLKs), whose structural organization and mode of activation are comparable with those of animal receptor tyrosine kinases (RTKs) (Box 1). For activation, plant RLKs require ligand-induced oligomerization followed by the recruitment of receptor-like cytoplasmic kinases (RLCKs). In the receptor complex, RLCKs are phosphorylated by kinase domains of receptors, after which they dissociate and modulate the activity of their respective downstream targets (Liang and Zhou, 2018). For example, it was shown that RLCK *Botrytis*-induced kinase 1 (BIK1) interacts directly with several RLKs to regulate plant immune response, phytohormone signaling, or stress tolerance (Box 2A). Experimental data indicate that downstream targets of RLCKs comprise common signaling nodes, such as reactive oxygen species (ROS) production or mitogen-activated protein kinase (MAPK) cascades (Liang and Zhou, 2018).

Box 2. **Models of intracellular and intracellular/extracellular ABA perception**

**(A) Intracellular ABA perception**

Simplified ABA signaling in Arabidopsis guard cells: upon perception of ABA, OST1 is released from PP2C inhibition (1) and activated by autophosphorylation (2). It phosphorylates SLAC1 (3) and KAT1 (4) channels, which leads to anion efflux and inhibits potassium influx. Downstream events are phosphorylation of RBOHF (5) and the accumulation of H_2_O_2_ in the apoplast (6). At the same time, OST1 translocates to the nucleus (7) and phosphorylates transcription factors from different ABA-responsive families (8). This results in transcriptional reprogramming; for example, in response to ABA in guard cells, OST1-mediated phosphorylation of AKS1 results in monomerization and inhibition of KAT1 transcription (9).

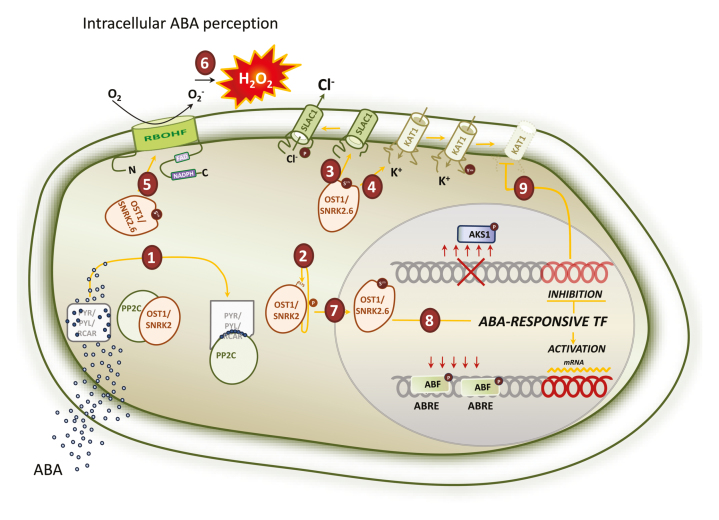

**(B) Membrane ABA perception**

There are still more questions than answers. The possible mechanism of RPK1-mediated ABA response can resemble pathogen detection (left part).

RPK1 can form a receptor complex with other membrane kinase-like receptors, BAK and CRKs.

In the beginning, ABA can be perceived intracellularly by the PYR/PYL/RCAR receptor; this results in the release of OST1 and RBOH activation (1) and accumulation of H_2_O_2_ in the apoplast (2). In turn, prolonged ABA accumulation can be perceived at the plasma membrane by detecting the changes in the apoplast redox poise by CRKs (3) and subsequent transphosphorylation of other RLKs in the receptor complex containing RPK1 (4), and resulted in the activation of downstream reactions (5). RPK1 activity in this receptor complex can be modulated by the basic ABA signaling pathway (OST1) (6). Alternatively, a specific ABA-binding membrane receptor may exist (7).

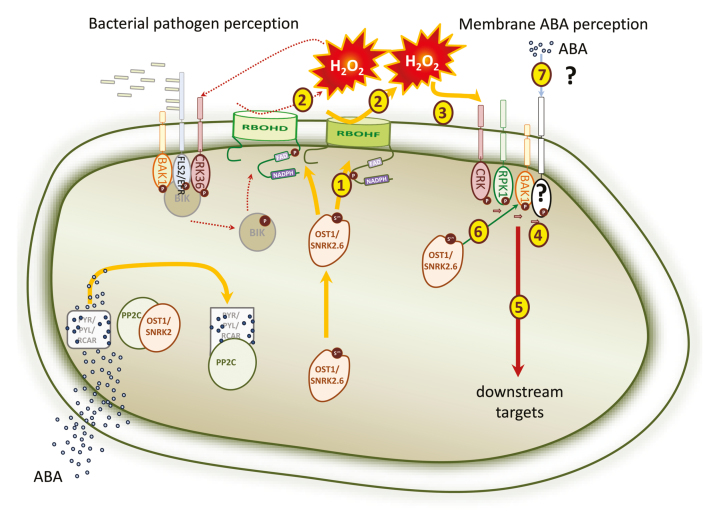

Some of the plant RLKs were already reported to be important for ABA signaling (Box 2B). Among them, besides receptor-like protein kinase 1 (RPK1), there are RLKs from the cysteine-rich family, CRKs (Tanaka *et al.*, 2012; Zhang *et al.*, 2013; Bourdais *et al.*, 2015; Lu *et al.*, 2016), proline-rich extensin-like receptor-like kinase RLK4 (PERK4) (Bai *et al.*, 2009), lectin receptor-like kinases (LecRK) (Deng *et al.*, 2009), GUARD CELL HYDROGEN PEROXIDE-RESISTANT 1 receptor-like kinase (GHR1) (Hua *et al.*, 2012), receptor DEAD-kinase 1 (RDK1) (Kumar *et al.*, 2017), and FERONIA receptor kinase (Yu *et al.*, 2012). Still, our knowledge about the cross-talk of membrane perception pathways with the basic ABA signaling pathway managed by OST1 remains limited. RLKs have either a positive or a negative effect on ABA signaling, and some of them were shown to interfere differently with the basic ABA-mediated pathway. For example, upon co-expression in *Xenopus laevis* oocytes, GHR1 interacts with SLAC1 (Hua *et al.*, 2012). It can be assumed that it activates channel conductance by phosphorylation. The activation was inhibited by phosphatase ABI2 but not by ABI1. In turn, RDK1 was shown to mediate recruitment of ABI1 to the plasma membrane (Kumar *et al.*, 2017).

## Interaction between membrane RPK1 and the basic intracellular ABA pathway

In this issue, Shang *et al.* (2020) analyse the molecular mechanism of RPK1 activity in ABA-induced stomatal closure and its possible interaction with the basic ABA-mediated pathway. An initial experiment showed that similarly to BAK1, RPK1 functions in the guard cells upstream of OST1. BAK1 itself does not bind a ligand but functions as a co-receptor, and its activity is critical for the formation of multimeric complexes, with many (if not all) RLKs involved in a wide range of physiological processes, such as immunity, growth, or development. Thus, it can be assumed that upon ABA-induced stomatal closure, membrane receptor RPK1 recruits BAK1 and OST1 to the common complex near the plasma membrane, in which BAK1 functions as a master regulator while OST1 functions in a similar manner to RLCK. The authors show that upon ABA stimulation RPK1 forms complexes with both BAK1 and OST1 but with different kinetics. Based on the gene expression, and two techniques of monitoring of *in vivo* complex formation (fluorescence resonance energy transfer and multicolour bimolecular fluorescence complementation), the authors conclude that BAK1–OST1 is created first from pre-existing proteins (with a peak at 30 min after ABA treatment), whereas the RPK1–OST1 complex is formed from the *de novo* synthesized protein later on (a gradual increase between 30 min and 2 h after ABA). Finally, the authors find that phosphatase ABI1 that regulates OST1 activity in the shortest basic ABA signaling pathway can also dephosphorylate RPK1, in a time-dependent manner, but not BAK1. RPK1 directly interacts with OST1, regulating phosphorylation and affecting the activity of the latter.

Overall, the results of this study indicate that during stomatal closure, RPK1-mediated response converges with the shortest basic ABA signaling pathway on the level of OST1 activation. However, in such a scenario, an important question remains open: the molecular mechanisms of RPK1 activation upon ABA treatment. To activate kinase activity of RLKs they have to oligomerize upon ligand binding. Therefore, it is tempting to speculate that, with the action of ABA, the RPK1 receptor at a plasma membrane becomes organized in multiprotein complex/complexes with BAK1 and redox-activated ABA-responsive CRK, similarly to FLAGELLIN SENSING 2 (FLS2), EFRs, and BAK1 during the pathogen-induced response. Such oligomerization may be induced by H_2_O_2_ accumulation in the apoplast resulting from OST1-mediated activation of RBOHF (Box 2). This concept sheds new light on our understanding of cross-talk among membrane-perceived and intracellular plant signaling pathways.

## References

[CIT0001] Acharya BR, Jeon BW, Zhang W, Assmann SM 2013 Open Stomata 1 (OST1) is limiting in abscisic acid responses of Arabidopsis guard cells. New Phytologist 200, 1049–1063. 24033256 10.1111/nph.12469

[CIT0002] Arnaud D, Hwang I 2015 A sophisticated network of signaling pathways regulates stomatal defenses to bacterial pathogens. Molecular Plant 8, 566–581. 25661059 10.1016/j.molp.2014.10.012

[CIT0003] Bai L, Zhang G, Zhou Y, Zhang Z, Wang W, Du Y, Wu Z, Song C-P 2009 Plasma membrane-associated proline-rich extensin-like receptor kinase 4, a novel regulator of Ca signalling, is required for abscisic acid responses in *Arabidopsis thaliana*. The Plant Journal 60, 314–327. 19566594 10.1111/j.1365-313X.2009.03956.x

[CIT0004] Bourdais G, Burdiak P, Gauthier A, et al 2015 Large-scale phenomics identifies primary and fine-tuning roles for CRKs in responses related to oxidative stress. PLoS Genetics 11, e1005373. 26197346 10.1371/journal.pgen.1005373 PMC4511522

[CIT0005] Cutler SR, Rodriguez PL, Finkelstein RR, Abrams SR 2010 Abscisic acid: emergence of a core signaling network. Annual Review of Plant Biology 61, 651–679. 10.1146/annurev-arplant-042809-112122 20192755

[CIT0006] Deng K, Wang Q, Zeng J, Guo X, Zhao X, Tang D, Liu X 2009 A lectin receptor kinase positively regulates ABA response during seed germination and is involved in salt and osmotic stress response. Journal of Plant Biology 52, 493.

[CIT0007] Harris JM 2015 Abscisic acid: hidden architect of root system structure. Plants 4, 548–572. 27135341 10.3390/plants4030548 PMC4844405

[CIT0008] Hua D, Wang C, He J, Liao H, Duan Y, Zhu Z, Guo Y, Chen Z, Gong Z 2012 A plasma membrane receptor kinase, GHR1, mediates abscisic acid- and hydrogen peroxide-regulated stomatal movement in Arabidopsis. The Plant Cell 24, 2546–2561. 22730405 10.1105/tpc.112.100107 PMC3406912

[CIT0009] Kimura S, Waszczak C, Hunter K, Wrzaczek M 2017 Bound by fate: the role of reactive oxygen species in receptor-like kinase signaling. The Plant Cell 29, 638–654. 28373519 10.1105/tpc.16.00947 PMC5435433

[CIT0010] Koo JC, Lee IC, Dai C, et al 2017 The protein trio RPK1–CaM4–RbohF mediates transient superoxide production to trigger age-dependent cell death in Arabidopsis. Cell Reports 21, 3373–3380. 29262318 10.1016/j.celrep.2017.11.077

[CIT0011] Kumar D, Kumar R, Baek D, Hyun T-K, Chung WS, Yun D-J, Kim J-Y 2017 *Arabidopsis thaliana* RECEPTOR DEAD KINASE1 functions as a positive regulator in plant responses to ABA. Molecular Plant 10, 223–243. 27923613 10.1016/j.molp.2016.11.011

[CIT0012] Lee DS, Kim YC, Kwon SJ, Ryu C-M, Park OK 2017 The Arabidopsis cysteine-rich receptor-like kinase CRK36 regulates immunity through interaction with the cytoplasmic kinase BIK1. Frontiers in Plant Science 8, 1856. 29163585 10.3389/fpls.2017.01856 PMC5663720

[CIT0013] Liang X, Zhou J-M 2018 Receptor-like cytoplasmic kinases: central players in plant receptor kinase-mediated signaling. Annual Review of Plant Biology 69, 267–299. 10.1146/annurev-arplant-042817-040540 29719165

[CIT0014] Lu K, Liang S, Wu Z, Bi C, Yu Y-T, Wang X-F, Zhang D-P 2016 Overexpression of an Arabidopsis cysteine-rich receptor-like protein kinase, CRK5, enhances abscisic acid sensitivity and confers drought tolerance. Journal of Experimental Botany 67, 5009–5027. 27406784 10.1093/jxb/erw266 PMC5014153

[CIT0015] Nambara E, Marion-Poll A 2005 Abscisic acid biosynthesis and catabolism. Annual Review of Plant Biology 56, 165–185. 10.1146/annurev.arplant.56.032604.144046 15862093

[CIT0016] Ng LM, Melcher K, Teh BT, Xu HE 2014 Abscisic acid perception and signaling: structural mechanisms and applications. Acta Pharmacologica Sinica 35, 567–584. 24786231 10.1038/aps.2014.5 PMC4813750

[CIT0017] Osakabe Y, Mizuno S, Tanaka H, et al 2010 Overproduction of the membrane-bound receptor-like protein kinase 1, RPK1, enhances abiotic stress tolerance in Arabidopsis. Journal of Biological Chemistry 285, 9190–9201. 20089852 10.1074/jbc.M109.051938 PMC2838338

[CIT0018] Racolta A, Nodine MD, Davies K, Lee C, Rowe S, Velazco Y, Wellington R, Tax FE 2018 A common pathway of root growth control and response to CLE peptides through two receptor kinases in Arabidopsis. Genetics 208, 687–704. 29187505 10.1534/genetics.117.300148 PMC5788531

[CIT0019] Shang Y, Yang D, Ha Y, Shin H-Y, Nam KH 2020 RPK1 and BAK1 sequentially form complexes with OST1 to regulate ABA-induced stomatal closure. Journal of Experimental Botany 71, 1491–1502. 10.1093/jxb/erz489 31665747

[CIT0020] Shi C-C, Feng C-C, Yang M-M, Li J-L, Xiao-Xu Li, Zhao B-C, Huang Z-J, Ge R-C 2014 Overexpression of the receptor-like protein kinase genes AtRPK1 and OsRPK1 reduces the salt tolerance of *Arabidopsis thaliana*. Plant Science 217–218, 63–70. 10.1016/j.plantsci.2013.12.002 24467897

[CIT0021] Shinozawa A, Otake R, Takezawa D, et al 2019 SnRK2 protein kinases represent an ancient system in plants for adaptation to a terrestrial environment. Communications Biology 2, 30. 30675528 10.1038/s42003-019-0281-1 PMC6340887

[CIT0022] Tanaka H, Osakabe Y, Katsura S, Mizuno S, Maruyama K, Kusakabe K, Mizoi J, Shinozaki K, Yamaguchi-Shinozaki K 2012 Abiotic stress-inducible receptor-like kinases negatively control ABA signaling in Arabidopsis. The Plant Journal 70, 599–613. 22225700 10.1111/j.1365-313X.2012.04901.x

[CIT0023] Wang H, Chevalier D, Larue C, Ki Cho S, Walker JC 2007 The protein phosphatases and protein kinases of *Arabidopsis thaliana*. The Arabidopsis Book 5, e0106. 22303230 10.1199/tab.0106 PMC3243368

[CIT0024] Wasilewska A, Vlad F, Sirichandra C, Redko Y, Jammes F, Valon C, Frei dit Frey N, Leung J 2008 An update on abscisic acid signaling in plants and more… Molecular Plant 1, 198–217. 19825533 10.1093/mp/ssm022

[CIT0025] Yang W, Zhang W, Wang X 2017 Post‐translational control of ABA signalling: the roles of protein phosphorylation and ubiquitination. Plant Biotechnology Journal 15, 4–14. 27767245 10.1111/pbi.12652 PMC5253474

[CIT0026] Yu F, Qian L, Nibau C, et al 2012 FERONIA receptor kinase pathway suppresses abscisic acid signaling in Arabidopsis by activating ABI2 phosphatase. Proceedings of the National Academy of Sciences, USA 109, 14693–14698. 10.1073/pnas.1212547109 PMC3437822 22908257

[CIT0027] Zhang X, Yang G, Shi R, Han X, Qi L, Wang R, Xiong L, Li G 2013 Arabidopsis cysteine-rich receptor-like kinase 45 functions in the responses to abscisic acid and abiotic stresses. Plant Physiology and Biochemistry 67, 189–198. 23583936 10.1016/j.plaphy.2013.03.013

